# Expression profiling of marker genes responsive to the defence-associated phytohormones salicylic acid, jasmonic acid and ethylene in *Brachypodium distachyon*

**DOI:** 10.1186/s12870-016-0749-9

**Published:** 2016-03-02

**Authors:** Yusuke Kouzai, Mamiko Kimura, Yurie Yamanaka, Megumi Watanabe, Hidenori Matsui, Mikihiro Yamamoto, Yuki Ichinose, Kazuhiro Toyoda, Yoshihiko Onda, Keiichi Mochida, Yoshiteru Noutoshi

**Affiliations:** Graduate School of Environmental and Life Science, Okayama University, Kita-ku Okayama, Japan; Cellulose Production Research Team, Biomass Engineering Research Division, RIKEN Center for Sustainable Resource Science, Tsurumi Yokohama, Japan

**Keywords:** *Brachypodium distachyon*, Phytohormone, Salicylic acid, Jasmonic acid, Ethylene, Plant disease resistance, Defense mechanism, Immunity system, Marker gene

## Abstract

**Background:**

*Brachypodium distachyon* is a promising model plants for grasses. Infections of *Brachypodium* by various pathogens that severely impair crop production have been reported, and the species accordingly provides an alternative platform for investigating molecular mechanisms of pathogen virulence and plant disease resistance. To date, we have a broad picture of plant immunity only in *Arabidopsis* and rice; therefore, *Brachypodium* may constitute a counterpart that displays the commonality and uniqueness of defence systems among plant species. Phytohormones play key roles in plant biotic stress responses, and hormone-responsive genes are used to qualitatively and quantitatively evaluate disease resistance responses during pathogen infection. For these purposes, defence-related phytohormone marker genes expressed at time points suitable for defence-response monitoring are needed. Information about their expression profiles over time as well as their response specificity is also helpful. However, useful marker genes are still rare in *Brachypodium*.

**Results:**

We selected 34 candidates for *Brachypodium* marker genes on the basis of protein-sequence similarity to known marker genes used in *Arabidopsis* and rice. *Brachypodium* plants were treated with the defence-related phytohormones salicylic acid, jasmonic acid and ethylene, and their transcription levels were measured 24 and 48 h after treatment. Two genes for salicylic acid, 7 for jasmonic acid and 2 for ethylene were significantly induced at either or both time points. We then focused on 11 genes encoding pathogenesis-related (PR) 1 protein and compared their expression patterns with those of *Arabidopsis* and rice. Phylogenetic analysis suggested that *Brachypodium* contains several *PR1*-family genes similar to rice genes. Our expression profiling revealed that regulation patterns of some *PR1* genes as well as of markers identified for defence-related phytohormones are closely related to those in rice.

**Conclusion:**

We propose that the *Brachypodium* immune hormone marker genes identified in this study will be useful to plant pathologists who use *Brachypodium* as a model pathosystem, because the timing of their transcriptional activation matches that of the disease resistance response. Our results using *Brachypodium* also suggest that monocots share a characteristic immune system, defined as the common defence system, that is different from that of dicots.

**Electronic supplementary material:**

The online version of this article (doi:10.1186/s12870-016-0749-9) contains supplementary material, which is available to authorized users.

## Background

To counteract various pathogens in the field, plants mainly protect themselves with a two-layered immune system. Using cell surface-localised receptors, plants recognise pathogen- or microbe-associated molecular patterns (PAMPs or MAMPs), which are structurally conserved molecules in a broad range of microorganisms, that may include products of housekeeping genes or cell wall components and induce the expression of defence-related genes. This system provides basal resistance called PAMP/MAMP-triggered immunity (PTI/MTI) [1]. For the successful infection of host plants, pathogens use a few dozen effector proteins as a weapon to suppress PTI. Plants can directly or indirectly sense these effectors by cytoplasmic nucleotide-binding domain- and leucine-rich repeat-containing (NLR) immune sensors and activate a strong resistance response called effector-triggered immunity (ETI) that is effective against pathogens [2]. ETI is often accompanied by hypersensitive responses including programmed cell death of infected regions containing pathogens. In a battery of these immune responses, the phytohormone salicylic acid (SA) plays important roles in mediating signal transduction. Another phytohormone, ethylene (ET), is also required to maintain the level of pattern-recognition receptors in PTI [3]. This defence system effectively functions to block biotrophic or hemibiotrophic pathogens. Plants have another defence system relying on the phytohormones jasmonic acid (JA) and ET to combat necrotrophic pathogens and insects [4].

To characterise plant responses to a given pathogen, the production of phytohormones may be appropriate indicators in addition to the phenotypic observation of lesion formation. However, in rice and barley, endogenous SA levels are not increased, even in response to incompatible pathogens, unlike the case of well-studied dicotyledonous model plants such as *Arabidopsis thaliana* and tobacco [5–7]. Alternatively, phytohormone production can be substituted with the expression profiling of phytohormone-responsive marker genes. This approach provides information about the time, strength and kind of responses provoked in plants. For example, *PATHOGENESIS-RELATED1* (*PR1*) and *PDF1.2* (*PLANT DEFENSIN1.2*) are used as markers for SA and JA or ET, respectively, in *Arabidopsis* [8, 9]. In model plants, genes considered to be involved in phytohormone biosynthesis or signalling are also used as markers [9, 10].

*Brachypodium distachyon* (purple false brome) is a grass plant of the Pooideae subfamily, which includes economically important crops such as wheat, barley, rye and oats. Owing to its small stature, short lifecycle, self-fertility and small diploid genome, *Brachypodium* can be an experimental model plant for studies of grasses including cereals and biomass crops [11]. A whole-genome sequence of *B. distachyon* cultivar Bd21 was obtained [12] and a database of full-length cDNA (FLcDNA) is available [13]. Recently, the superiority of this plant as a model for Triticeae crops has been shown by the similarities of morphological property and by the commonalities of metabolic profile [14]. For investigation of immunity as one of the important traits in agriculture, infectivity on *Brachypodium* of various pathogens threatening world crop cultivation has been verified so far [15]. For example, *Fusarium graminearum* and *Magnaporthe oryzae*, causal fungi of wheat *Fusarium* head blight and rice blast, respectively, are pathogenic to *Brachypodium* [16, 17]. Bacterial pathogen *Xanthomonas oryzae* pv. *oryzae* and a pathogenic virus *Panicum mosaic virus* are also virulent to *Brachypodium* [18, 19]. Thus, *Brachypodium* may be a useful platform for investigating both crop pathogen virulence and plant immune response at the molecular level.

Several phytohormone marker genes have been used to date to characterise resistance responses in *Brachypodium*, but the number of markers is still limited and inadequate. Most recently, a comprehensive transcriptome analysis of various phytohormones in *Brachypodium* using RNA-seq technology was performed and phytohormone-responsive genes were identified [20]. In that study, hormone treatment was for 1 h for JA and ET and 3 h for SA using young seedlings. For investigations of plant–microbe interaction, for each immune phytohormone, several sets of marker genes up-regulated at appropriate time points during infection process are needed.

For the present study, we chose candidates for *Brachypodium* genes responsive to SA, JA and ET based on the similarity of protein sequences to known marker genes used in *Arabidopsis* and rice and analysed their transcriptional activation by each hormone at 24 and 48 h after treatment. As a result, we identified at least 2 marker genes for each hormone. In addition, we compared the constitutions and expression profiles of *PR1* family genes from *Arabidopsis*, rice and *Brachypodium*, finding that *B. distachyon* possesses immunity mechanisms similar to those of rice but not of *Arabidopsis*.

## Results and discussions

### Identification of candidates for marker genes responsive to defence-related phytohormones in *Brachypodium*

We selected candidates for phytohormone-responsive genes in *Brachypodium,* based on the similarities to experimentally validated markers in rice, barley and *Arabidopsis*. For *BdTARL1* and *BdTARL2* genes in *B. distachyon*, their responsiveness to 1-aminocyclopropane-1-carboxylic acid (ACC), a precursor of ET, has already been demonstrated [21]. The protein sequences of these selected genes were used as queries in a BLAST search against the RIKEN *Brachypodium* FLcDNA database, and the resulting hits with high similarity were identified as potential markers [13, 22]. Twenty-three genes were tested for transcriptional inductions during treatment with SA, JA or ET (Table 1).

**Table 1:** Candidate marker genes selected in this study for SA, JA and ET in *Brachypodium*

ID	Name	Description in database	Rice homolog	Arabidopsis homolog	Ref
SA-related genes					
*Bradi2g05870*	NPR1	Regulatory protein NPR1-like	*OsNPR1:Os01g0194300*	*NPR1 : At1g64280*	[24]
*Bradi2g30695*	WRKY45-1	Uncharacterized protein	*OsWRKY45-1* : *Os05g0322900*	*AtWRKY70 : At3g56400*	[24]
*Bradi2g44270*	WRKY45-2	WRKY transcription factor 70-like	*OsWRKY45-1* : *Os05g0322900*	*AtWRKY70 : At3g56400*	[24]
*Bradi4g35356*	SAGT1	UDP-glycosyltrasferase 74 F1-like	*OsSGT1* : *Os09g0518200*	*UGT superfamily : At1g05675*	[29]
*Bradi2g22410*	AGA	Alanine-glyoxylate aminotransferase 2 homolog 3	*Osh36* : *Os05g0475400*	*AtPYD4 : At3g08860*	[29]
*Bradi1g53527*	UGT76-1	UDP-glycosyltrasferase 76C2-like	no symbol : *Os07g0241500*	*UGT76B1 : At3g11340*	[30]
*Bradi1g53540*	UGT76-2	UDP-glycosyltrasferase 76C2-like	no symbol : *Os07g0241500*	*UGT76B1 : At3g11340*	[30]
*Bradi1g53550*	UGT76-3	UDP-glycosyltrasferase 76 F1-like	no symbol : *Os07g0241500*	*UGT76B1 : At3g11340*	[30]
*Bradi4g41410*	UGT76-4	UDP-glycosyltrasferase 76C2-like	no symbol : *Os07g0241500*	*UGT76B1 : At3g11340*	[30]
*Bradi1g11940*	UGT74-1	Indole-3-acetate beta-glucosyltransferase-like	*OsIAGLU* : *Os03g0693600*	*UGT74F2 : At2G43820*	[30]
*Bradi4g35350*	UGT74-2	UDP-glycosyltrasferase 74 F2-like	no symbol : *Os09g0517900*	*UGT74F2 : At2G43820*	[30]
*Bradi5g03380*	UGT74-3	UDP-glycosyltrasferase 74 F2-like	no symbol : *Os04g0206500*	*UGT74F2 : At2G43820*	[30]
JA-related genes					
*Bradi1g69330*	AOS	Allene oxide synthase 2-like	*OsAOS2 : Os03g0225900*	*AtAOS2 : At5g42650*	[32–34]
*Bradi1g11670*	LOX	Linoleate 9S-lipoxygenase 4-like	*OsLOX1 : Os03g0700700*	*AtLOX5 : At3g22400*	[32–34]
ET-related genes					
*Bradi2g52370*	ERF	Ethylene-responsive transcription factor 4-like	*OsERF3 : Os01g0797600*	*AtERF9 : At5g44210*	[43]
*Bradi1g63780*	EIN3	Ethylene insensitive 3-like	no symbol : *Os03g0324300*	*AtEIN3 : At3g20770*	[42]
*Bradi1g49966*	ACC	Aminotransferase ACS10-like	*OsACS6 : Os06g0130400*	*AtACS10 : At1g62960*	[44]
*Bradi2g34400*	TAR1	Tryptophan aminotransferase-related protein 2-like	*OsTAR1 : Os05g0169300*	*AtTAR2 : At4g24670*	[21]
*Bradi2g04290*	TAR2	Tryptophan aminotransferase-related protein 2-like	*OsTAR1 : Os05g0169300*	*AtTAR2 : At4g24670*	[21]
*Bradi3g37300*	4CL	4-Coumarate:CoA ligase 5-like	*Os4CL5 : Os08g0448000*	*At4CL1 : At1g51680*	[35, 37–39]
*Bradi3g48840*	PAL	Phenylalanine ammonia-lyase-like	*OsPAL1 : Os02g0627100*	*AtPAL1 : At2g37040*	[35, 37–39]
*Bradi1g33540*	PR5	Thaumatin-like protein-like	no symbol : *Os06g0691200*	no symbol : *At1g73620*	[45, 47]
*Bradi4g05040*	PBZ1	Major allergen Api g 1-like	*PBZ1-like : Os12g0555000*	no hit	[45, 47]

Twenty-three *Brachypodium* genes were identified by similarity search using known phytohormone marker genes of rice or *Arabidopsis* as queries. Gene IDs, relationships to phytohormone, expedient names without functional confirmation, descriptions in the database, corresponding homologs in rice or *Arabidopsis*, and references are listed

Whole *Brachypodium* seedlings were treated with water as a mock treatment, 1 mM sodium salicylate, 100 μM methyl jasmonate (MeJA) or 100 μM ethephon for 24 or 48 h. Total RNAs were extracted from the frozen leaf samples and subjected to cDNA synthesis. The mRNA levels of the candidate genes were analysed by quantitative reverse-transcription polymerase chain reaction (qRT-PCR) using specific primers designed with the Primer3 program [23]. The responsiveness of each gene is summarised in Table 2. Among these genes, 8 were significantly induced by a phytohormone, whereas the remaining 15 genes showed no change in expression.

**Table 2:** Transcriptional responses of tested genes to SA, JA and ET

		Inducibility in *Brachypodium*		
ID	Name	SA	JA	ET
*Bradi2g05870*	*NPR1*	-	-	-
*Bradi2g30695*	*WRKY45-1*	++	-	-
*Bradi2g44270*	*WRKY45-2*	++	+ (48 h)	+ (48 h)
*Bradi4g35356*	*SAGT1*	-	-	-
*Bradi2g22410*	*AGA*	-	-	-
*Bradi1g53527*	*UGT76-1*	-	-	-
*Bradi1g53540*	*UGT76-2*	-	-	-
*Bradi1g53550*	*UGT76-3*	-	-	-
*Bradi4g41410*	*UGT76-4*	-	-	+
*Bradi1g11940*	*UGT74-1*	-	-	-
*Bradi4g35350*	*UGT74-2*	-	-	-
*Bradi5g03380*	*UGT74-3*	-	-	-
*Bradi1g69330*	*AOS*	-	++	-
*Bradi1g11670*	*LOX*	-	+	-
*Bradi2g52370*	*ERF*	-	-	-
*Bradi1g63780*	*EIN3*	-	-	-
*Bradi1g49966*	*ACC*	-	-	-
*Bradi2g34400*	*TAR1*	-	-	-
*Bradi2g04290*	*TAR2*	-	-	+
*Bradi3g37300*	*4CL*	+ (48 h)	++	+ (48 h)
*Bradi3g48840*	*PAL*	-	++	-
*Bradi1g33540*	*PR5*	-	-	-
*Bradi4g05040*	*PR10(PBZ1)*	-	-	-

Expression of 23 *Brachypodium* candidate genes was evaluated in 3–4 week-old plants at 24 and 48 h after treatment with SA, JA or ET, and the results are summarised. The expression levels of each gene were determined by qRT-PCR analysis. ++, genes significantly induced more than 10-fold compared to mock treatment; +, genes significantly induced more than 2-fold compared to mock treatment, −, not induced. Experiments were performed at least three times with similar results and a representative result is shown

**Fig. 1:**
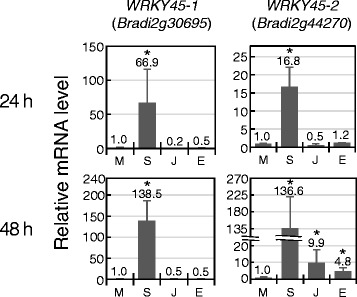
Expression patterns of SA-responsive genes. Expression levels of *WRKY45-1*(*Bradi2g30695*) and *WRKY45-2*(*Bradi2g44270*) were determined by qRT-PCR analyses at 24 (*upper panel*) or 48 h (*lower panel*) after treatment with the indicated phytohormones. Data are presented as means of relative expression values of three independent treatments compared to mock treatment. M, mock treatment; S, SA treatment; J, JA treatment; E, ET treatment. Error bars represent standard error (*n* = 3). Asterisks above the bars indicate significant differences compared to mock treatment at *P* < 0.05 (Student’s *t* test). Experiments were performed at least three times with similar results, and a representative result is shown

To obtain SA markers in *Brachypodium*, we focused on genes encoding WRKY-domain containing transcription factors. In rice, *OsWRKY45*, *62* and *76* genes were induced by SA treatment, and all of them were shown to participate in the immune response [24–26]. Among them, *OsWRKY45* plays a central role in SA signalling, together with *OsNPR1*, and mediates SA-induced disease resistance [24]. Using RNA-seq technology in rice, transcriptional upregulation of *OsWRKY45* was detected at 24 h after inoculation of both compatible and incompatible strains of *M. oryzae* [27]. Its induction by SA was also observed 12 h after SA treatment [24]. In *Brachypodium,* two genes, *Bradi2g30695* and *Bradi2g44270,* were found, whose deduced protein sequences showed high similarity (49 and 50 % identity, respectively) to OsWRKY45 throughout their lengths (Additional file 1: Figure S1). As shown in Fig. 1, transcription of these genes was upregulated by SA at 24 h after treatment and their expression levels were more increased at 48 h. Kakei et al. also reported that *Bradi2g44270* and *Bradi2g30695* were induced at 3 h after treatment with 100 μM SA [20]. For *Bradi2g44270*, 9.9- and 4.8-fold expression changes were also detected at 48 h following treatment with JA and ET, respectively, although their induction levels were lower than those with SA. OsWRKY62 and 76 are negative regulators of disease resistance responses in rice [25, 26], and no *Brachypodium* homologs for *OsWRKY62* were found, whereas three genes, *Bradi4g30360*, *Bradi1g30870* and *Bradi3g06070,* showed similarity to *OsWRKY76*. In the RNA-seq results by Kakei et al., only *Bradi4g30360*, the gene most similar to *OsWRKY76* among the *Brachypodium* homologs, was induced (with a log_2_ ratio of 3) at 3 h after SA treatment.

During disease resistance response in *Arabidopsis*, SA is biologically synthesized to induce defence responses and is subsequently metabolised to reset the immunity mode. One of the major SA metabolism pathways is glycosylation, in which SA glucosyltransferase (SAGT) conjugates a glucose moiety to SA to produce SA-O-β-D-glucoside (SAG) using UDP-glucose as a donor. SAG is an inactive form of SA [28]. In *Arabidopsis* and rice, SA treatment leads to increased expression of *SAGT* genes [29, 30]. Under the hypothesis that *SAGT* is an SA marker, *Brachypodium SAGT* genes were retrieved from the cDNA database. Four and three *Brachypodium* homologs of *Arabidopsis* UGT76B1 and UGT74F1, respectively, showing identities of > 40 % in their amino acid sequences, were identified. One homolog with the highest similarity to OsSGT1 was also selected. In *Brachypodium*, no induction by SA was detected for these 7 *SAGT* genes (Table 2). Instead, we found that *Bradi4g41410* was induced by ET (Fig. 3). It is not clear whether the genes used in this study function as SAGT, given that more than 170 predicted UGT genes were found in the *Brachypodium* genome and sequence similarity using whole length does not always reflect functional identity. Other studies are needed to identify the players involved in SA metabolism in *Brachypodium*.

**Fig. 2:**
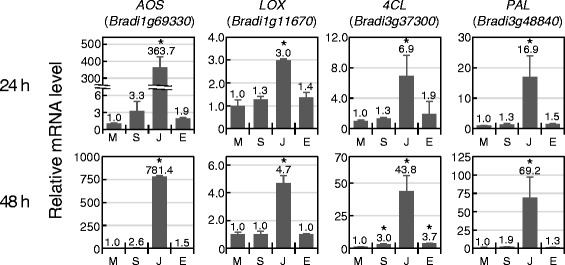
Expression patterns of JA-responsive genes. Expression levels of two JA-inducible genes at 24 h (*upper panel*) or 48 h (*lower panel*) after treatment with phytohormones. Transcript levels were determined by qRT-PCR analyses, and relative expression levels compared to mock treatment are presented. M, mock treatment; S, SA treatment; J, JA treatment; E, ET treatment. Error bars represent standard error (*n* = 3 independent treatments). Asterisks above the bars indicate significant differences compared to mock treatment at *P* < 0.05 (Student’s *t* test). The experiment was performed at least three times with similar results, and a representative result is shown

Allene oxide synthase (AOS) and lipoxygenase (LOX) are required for JA biosynthesis [31]. Positive feedback regulation in transcription of these enzyme-encoding genes by JA is well understood and they are used as JA markers in various plant species. In *Arabidopsis*, expression of *AtAOS2* and *AtLOX2* were upregulated by JA [32]. In rice, induction of *OsAOS2* and *OsLOX1* was detected at 6 h after JA treatment, according to the rice global expression profile database RiceXPro [33]. In barley, JA responsiveness of *AOS* (contig3096_s_at) and *LOX* (contig2306_s_at) was validated by microarray analysis and semi-quantitative RT-PCR [34]. Four *Brachypodium* genes, *Bradi1g69330, Bradi1g07480, Bradi3g08160* and *Bradi3g01110,* were identified as homologs of *OsAOS2* by blastp search, and *Bradi1g69330*, with the highest score, was used in this study. Its deduced protein sequence also shows high similarity to barley AOS. We detected strong induction of this *Brachypodium AOS* gene at 24 h after JA treatment, and its level was doubled at the 48 h time point (Fig. 2). For *LOX*, 10 genes (*Bradi1g11670, Bradi1g11680, Bradi1g09260, Bradi1g09270, Bradi3g59710, Bradi5g11590, Bradi1g72690, Bradi3g39980, Bradi3g07010* and *Bradi3g07000*) were found as *OsLOX1* (*Os03g0700700*) homologs. The most similar *Bradi1g11670* gene has been shown to be expressed after infection by the fungal pathogen *Sclerotinia homeocarpa* in the resistant *Brachypodium* accession 208126 [35]. We accordingly checked its response to JA. As shown in Fig. 2, 3.0- and 4.7-fold expression changes were observed at 24 and 48 h, respectively, after hormone treatment. These results suggest that both genes would be useful JA markers.

During the disease resistance response, plants use phenylpropanoid compounds for the biosynthesis of lignin, flavonoids, and phytoalexins, which are required for the fortification of cell walls and production of antimicrobials [36]. 4-Coumarate:CoA ligase (4CL) and phenylalanine ammonia lyase (PAL) are key enzymes in this metabolic pathway, and the transcriptional upregulation of *PAL* and *4CL* after elicitor treatment and pathogen inoculation have been reported in *Arabidopsis*, rice and *Brachypodium* [35, 37–39]. In *Brachypodium*, three *4CL* homologs, *Bradi3g37300*, *Bradi3g05750* and *Bradi1g31320*, were identified by blastp search using the protein sequence of *Arabidopsis At1g51680* as a query (E value = 0). Similarly, *Bradi5g15830*, *Bradi3g48840*, *Bradi3g49280*, *Bradi3g49260*, *Bradi3g49270*, *Bradi3g47110*, *Bradi3g47120* and *Bradi3g49250* were found as homologs of *AtPAL1* (*At2g37040*). *Bradi3g37300* as a representative of *4CL* and *Bradi3g48840* for *PAL* were markedly induced at 24 h after JA treatment, with further-increased levels at 48 h (Fig. 2). We checked the expression of rice *OsPAL1* and *Os4CL5* using the RiceXPro database [33] and found that they were also induced within 6 h after JA treatment, in accord with our result. In our study, expression of *Brachypodium 4CL* was also detected by both SA and ET at 48 h. These *Brachypodium 4CL* and *PAL* genes have also been reported to be induced by JA (log_2_ ratio = 1.59 and 1.96, respectively) 1 h after 30 μM MeJA treatment [20].

**Fig. 3:**
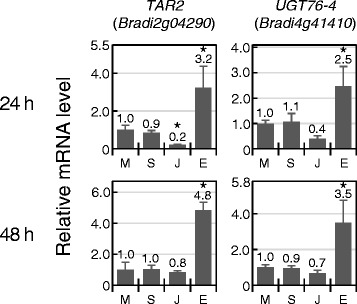
Expression patterns of ET-responsive genes. Expression levels of two ET-inducible genes at 24 h (*upper panel*) or 48 h (*lower panel*) after treatment with phytohormones. Transcript levels were determined by qRT-PCR analyses, and relative expression levels compared to mock treatment are presented. M, mock treatment; S, SA treatment; J, JA treatment; E, ET treatment. Error bars represent standard error (*n* = 3 independent treatments). Asterisks above the bars indicate significant differences compared to mock treatment at *P* < 0.05 (Student’s *t* test). The experiment was performed at least three times with similar results, and a representative result is shown

*Tryptophan aminotransferase of Arabidopsis 1 (TAA1)-related* (*TAR*) is required for the biosynthesis of indole-3-pyruvic acid from L-tryptophan in *Arabidopsis* [40] and its expression is upregulated by ET [41]. In *Brachypodium*, the expression levels of two *TAR* homologs, *BdTARL1* (*Bradi2g34400*) and *BdTARL2* (*Bradi2g04290*), have been shown to be increased at 3 h after ACC treatment (Table 2) [21]. Under our experimental conditions, transcription of *BdTARL2* but not *BdTARL1* was significantly induced at both 24 and 48 h after ethephon treatment (Fig. 3). *BdTARL2* may have been expressed continuously by ET from 3 to 48 h after the treatment. Because genes involved in biosynthesis and signalling of ET are often transcriptionally activated by ET in *Arabidopsis*, we selected *ACS* (*ACC SYNTHASE*) (*Bradi1g49966*), *ERF* (*ETHYLENE RESPONSIVE FACTOR*) (*Bradi2g52370*) and *EIN3* (*ETHYLENE-INSENSITIVE3*) (*Bradi1g63780*) as candidate ET-responsive genes. They were the closest homologs to the corresponding rice genes (Table 1) [42–44]. In our study, their transcription did not respond to ET (Table 2). In *Brachypodium*, we found a single homolog of *EIN3*, but there were 4 *ACS* homologs and over 100 homologs of *AP2/ERF* family genes. Thus, it is still possible that there are ET-responsive *ACS* and *ERF* in the genome. RNA-seq analysis at 3 h after ACC treatment identified only an *EIN4* homolog (*Bradi5g00700*) as an ET-responsive gene [20].

In rice, *pathogenesis-related* genes *PR5* and *PR10* (*PBZ1; PROBENAZOLE-INDUCED PROTEIN1*) are induced by ET or chitin, typical PAMPs [45, 46]. They belong to multigene families in rice, and we found 32 and 5 homologs in *Brachypodium* for *PR5* and *PR10*, respectively. The expression levels of *Bradi1g33540* and *Bradi4g05040* as marker candidates for *PR5* and *PR10*, respectively, were evaluated because they are the homologs most similar to *OsPR5* and *OsPR10*, and *Bradi1g33540* has already been shown to be induced by pathogens [19]. However, no induction by phytohormone treatment could be detected under our conditions (Table 2).

In summary, we successfully identified 2, 4 and 2 marker genes for SA, JA and ET, respectively. They may be useful tools for the characterisation of defence responses induced in *Brachypodium* in various host-parasite interactions.

### Characterisation of the phytohormone responsiveness of the *BdPR1* gene family in *Brachypodium*

SA is used for plant defence mainly against biotrophic pathogens, and JA and ET are mainly used against necrotrophic pathogens [47]. In *Arabidopsis*, SA and JA exert an antagonistic effect on each other [48]. For instance, SA treatment suppresses JA-inducible genes such as *PDF1.2*, *VSP1*, *LOX2*, *AOS*, *AOC2* and *OPR3* [49]. Recently, a genome-wide transcriptional analysis in rice using microarray revealed that more than half of 313 genes upregulated by benzothiadiazole (BTH), a functional analogue of SA, are also induced by JA, although a third of them were suppressed by JA [50]. This gene set, positively regulated by both SA and JA, is defined as a common defence system that is possibly used in response to various biotic and abiotic stresses in rice [50, 51]. *OsWRKY45* and several *OsPR1* genes are examples of genes belonging to this group with their expression levels increased by both SA and JA [52, 53].

On the other hand, this common defence system is not found in tobacco and *Arabidopsis.* In tobacco, PR1-family proteins consist of acidic and basic groups regulated by SA and JA, respectively, and the induction of each gene was antagonistically suppressed by the other hormones [54]. In *Arabidopsis*, only *AtPR1* (*At2g14610*) among 22 *PR1*-family genes is responsive to SA and pathogen inoculation based on microarray data [55], although *AtPRB1* was shown to be weakly induced by MeJA and ET in root [56]. These situations may depend on differences between rice and dicots in the SA signalling cascade [57]. We accordingly speculate that this common defence system is a characteristic feature of monocots. However, rice contains a high level of endogenous SA under normal conditions, unlike other monocots such as barley and *Brachypodium* [6, 58]. To determine whether this common defence system is specific to rice and arose during domestication or is shared by all monocots, we characterised the response nature of *PR1*-family genes in *Brachypodium* and compared it with those of rice and *Arabidopsis*.

A blastp search of the protein sequence of AtPR1 against the database of RIKEN *Brachypodium* FLcDNA clones, to identify *Brachypodium PR1* homologs, yielded 11 genes, defined as the *BdPR1* family, with high similarities in their deduced protein sequences (E value < 1E-10). Among them, 5 and 4 genes were located on chromosomes 1 and 3, respectively, and the remaining 2 genes were found on chromosomes 2 and 4. According to rice *PR1* gene nomenclature [52], these *BdPR1* genes were also designated based on their chromosomal locations. The order of precedence depends on both chromosome number and position from the 5′ end. For example, the 5 *BdPR1* members on chromosome 1 were named *BdPR1-1*, *BdPR1-2*, *BdPR1-3*, *BdPR1-4* and *BdPR1-5* in order from 5′ to 3′. The gene on chromosome 2 was named *BdPR1-6*.

**Fig. 4:**
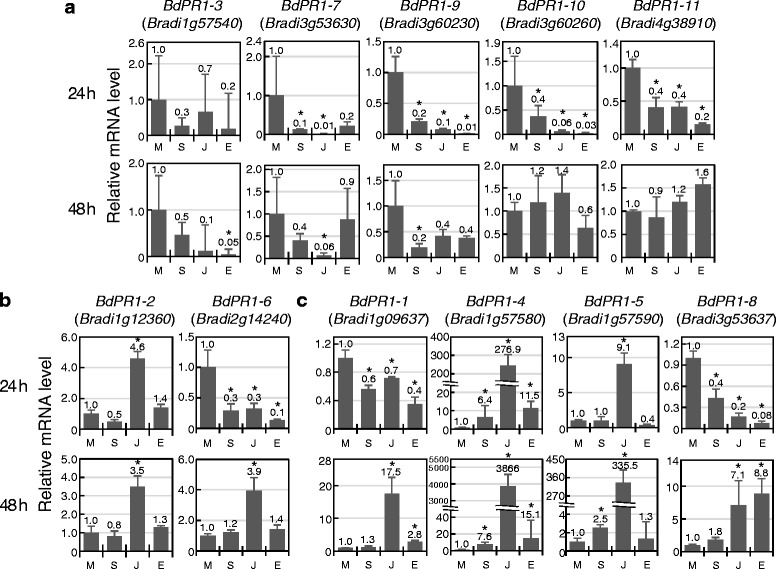
Expression patterns of *BdPR1* gene family after treatment with phytohormones. Expression levels of *BdPR1* genes at 24 or 48 h after phytohormone treatment were determined by qRT-PCR analyses. Transcript levels relative to those in mock treatment are presented. **a**, not inducible genes; **b**, genes only induced by JA; **c**, genes induced by multiple phytohormones. M, mock treatment; S, SA treatment; J, JA treatment; E, ET treatment. Error bars represent standard error (*n* = 3 independent treatments). Asterisks above the bars indicate significant differences compared to mock treatment at *P* < 0.05 (Student’s *t* test). The experiment was performed at least three times with similar results, and a representative result is shown

We designed primers for specific detection of each *BdPR1* gene in qRT-PCR experiments and evaluated their expressions at 24 and 48 h after treatment with SA, JA, or ET (Fig. 4). According to their expression patterns, *BdPR1* members were classified into three groups. Group A contains five *BdPR1* genes whose transcriptions were not upregulated by any phytohormone (Fig. 4a). Instead, their expressions were significantly or likely suppressed at 24 or 48 h after treatment with these phytohormones. Such suppression was similarly observed for *BdPR1-1*, *BdPR1-6* and *BdPR1-8*, which are categorised into other groups, at 24 h after phytohormone treatment. Two genes were in group B, members of which were responsive to only a single phytohormone, JA (Fig. 4b). *BdPR1-2* was induced at both 24 and 48 h, whereas *BdPR1-6* was upregulated only at 48 h. Group C comprises 4 genes induced by more than two phytohormones (Fig. 4c). Transcription of *BdPR1-1* and *BdPR1-8* was induced by JA and ET at 48 h after treatment. *BdPR1-5* expression responded to JA at 24 h and its level was further increased at 48 h. A weak response of this gene to SA was also detected at 48 h. As for *BdPR1-4*, its transcription was induced by all of the tested phytohormones. Its induction was especially sensitive to JA, and massive transcription was detected at 48 h.

**Fig. 5:**
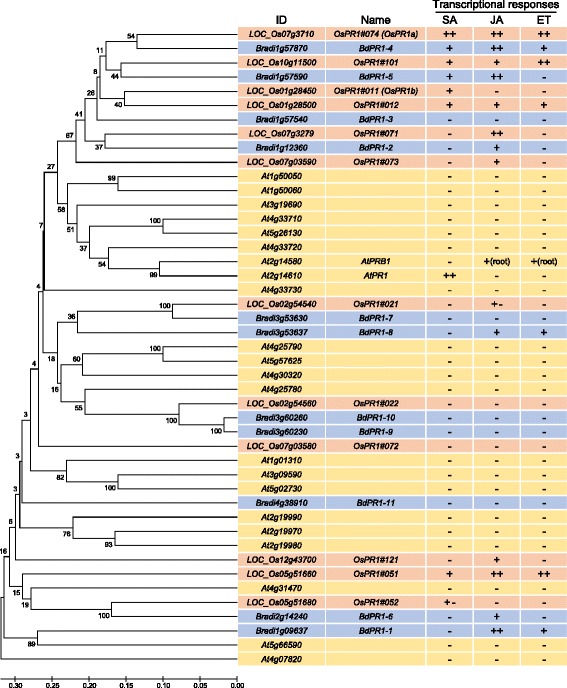
Phylogenetic analysis of *PR1* gene families in *Arabidopsis*, rice and *Brachypodium*. A phylogenetic tree of *PR1* gene families of *Arabidopsis*, rice and *Brachypodium* was constructed with MEGA software (http://www.megasoftware.net/) using the UPGMA method with bootstrap values (1000). Phytohormone inducibilities of *BdPR1* family analysed in this study and those of the *AtPR1* family and *OsPR1* family reported in van Loon et al. (2006) and Mitsuhara et al. (2008), respectively are summarised in the right column [52, 55]. Induction status is presented as follows: ++, significantly induced more than10-fold compared to the mock treatment; +, significantly induced more than 2-fold compared to the mock treatment; −, not inducible; +−, gene whose induction or expression was not clear

Our results revealed that some of the *Brachypodium PR1* genes were induced by multiple phytohormones, as reported in rice [52]. Using the predicted protein sequences of 11, 12 and 22 PR1 families of *Brachypodium*, rice and *Arabidopsis*, respectively, a phylogenetic tree was constructed by the UPGMA (Unweighted Pair Group Method with Arithmetic mean) method (Fig. 5). Protein sequences of the rice OsPR1 and the *Arabidopsis* AtPR1 family were obtained from the MSU Rice Genome Annotation Project and the Arabidopsis Information Resource (TAIR), respectively. The resulting tree illustrates that *Brachypodium* and rice contain similar sets of *PR1* family genes apart from *Arabidopsis*, and it suggests the difference between monocots and dicots in constitution of *PR1* family proteins. In the right columns of Fig. 5, we summarise the phytohormone responsiveness of these *Brachypodium PR1* genes as revealed in this study and the reported information for rice *OsPR1* and *Arabidopsis AtPR1* genes. In *AtPR1* genes, only two genes (*At4g25780*, *At5g66590*) were classified into the same clade of monocot *PR1* genes, whereas remaining 20 genes, which contained phytohormone responsive *AtPR1* and *AtPRB1*, formed independent clades. Some of the *PR1* genes from *Brachypodium* and rice classified into the same clade showed similar expression response patterns to the phytohormones. For example, *BdPR1-4* and *OsPR1#074* (*OsPR1a*) or *BdPR1-5* and *OsPR1#101* responded to multiple phytohormones, whereas *BdPR1-7, BdPR1-9*, *BdPR1-10, OsPR1#021* and *OsPR1#022* were not induced by any phytohormones. *BdPR1-2* and *OsPR1#071* were induced by only JA. Other gene pairs showed different expression patterns, suggesting different roles of the *PR1* family between these plant species.

From these situations, we hypothesized that a common defence system is present in *Brachypodium* and that the system is conserved among monocot plants. This idea is also supported by our findings that at least *WRKY45-2*, *4CL*, *BdPR1-4* and *BdPR1-5* were regulated by both SA and JA (Figs. 1, 2 and 4c). A comprehensive transcriptome analysis of *Brachypodium* using RNA-seq or microarrays may confirm this hypothesis.

## Conclusions

Genome deciphering by next-generation sequencing and comprehensive transcriptome analysis with RNA-seq enable comparative genomics in many crop species. Distinctive features in crops often impede the progress of detailed molecular analysis, but a large picture of plant immunity is available only in *Arabidopsis* and rice at present. Given that *Brachypodium* has attractive advantages that can overcome the limitations of crop research especially for Pooideae crops attributed to slow growth speed, large genome size, high ploidy and so on, it is expected to provide knowledge bearing on the commonality or uniqueness of defence systems among plant species. In this study, we identified the phytohormone marker genes *WRKY45-1* and *WRKY45-2* for SA; *AOS*, *LOX*, *4CL, PAL, PR1-2, PR1-5* and *PR1-6* for JA and *TAR* and *UGT76-4* for ET (Figs. 1, 2, 3 and 4). Having been selected for responsiveness on the bases of both time point and intensity, which are parameters used for monitoring plant reactions during infection by many phytopathogens, these genes should be useful tools not only for describing spatiotemporal immune responses to specific pathogens in *Brachypodium* but also for comparing them with those to other pathogens in a unified framework. The comparison of expression profiles of *PR1* family genes suggests that *Brachypodium* has phytohormone responses more similar to those of rice than of *Arabidopsis*.

## Methods

### Plant materials and growth conditions

The *Brachypodium distachyon* cultivar Bd21 was used. *Brachypodium* seeds were germinated on moist filter paper. After 7 days, the seedlings were transferred to wells of 24-well microtiter plates filled with soil and grown in a growth chamber (LPH-350S; Nippon Medical & Chemical Instruments, Osaka, Japan) at 23 °C under a 20 h light/4 h dark photoperiod [13].

### Phytohormone treatment

Sodium salicylate (SA; Wako, Osaka, Japan), MeJA (JA; Wako, Osaka, Japan) and ethephon (Sigma-Aldrich, St. Louis, MO, USA), an ET generator, were used as phytohormones. Whole Bd21 seedlings grown for 3 to 4 weeks were immersed in water (mock treatment) or a plant hormone solution (1 mM SA, 100 μl MeJA, or 100 μM ethephon) using 50-mL conical tubes. The seedlings were incubated for 24 or 48 h at 23 °C under a 20 h light/4 h dark photoperiod. Then, the first and second fully expanded leaves from the top of the seedlings were collected in 2-mL tubes and frozen in liquid nitrogen.

### RNA extraction and gene expression analysis

The frozen samples were crushed with four zirconia beads (ø 2 mm) using a Shake Master Neo (BMS, Tokyo, Japan). Total RNA was extracted with a Total RNA Purification Kit (JenaBioscience, Jena, Germany) with on-column DNase treatment (Invitrogen, Carlsbad, CA, USA). RNA concentration and purity were validated with a DS-11 spectrophotometer (Denovix, Wilmington, DE, USA). cDNA was synthesized from each sample with the PrimeScript RT reagent kit with gDNA Eraser (Takara, Shiga, Japan). Gene expression analyses were performed by qRT-PCR using a KAPA SYBR Fast qPCR Kit (KAPA BIOSYSTEMS, Woburn, MA, USA) with a GVP-9600 real-time PCR instrument (Shimadzu, Kyoto, Japan). The quantification of target transcripts was performed using the GVP-9600 internal software GVP gene detection system, and the data were normalised to the *BdUbi4* gene (*Bradi3g04730*), which has been established as a reference gene for expression studies in *B. distachyon* [59]. Primers used in this study are listed in Additional file 2: Table S1.

## Availability of data and materials

All supporting data can be found within the manuscript and its additional files.

## Additional files

Additional file 1: Figure S1. Protein sequence alignments of OsWRKY45, BdWRKY45-1 and BdWRKY45-2 (PPTX 145 kb) Additional file 2: Table S1. Primers used in this study (DOCX 32 kb)

## Abbreviations

ACC: 1-aminocyclopropane-1-carboxylic acid
ACS: ACC synthase
AOC: allene oxide cyclase
AOS: allene oxide synthase
BTH: benzothiadiazole
CoA: coenzyme A
EIN: ethylene insensitive
ERF: ethylene responsive factor
ET: ethylene
ETI: effector-triggered immunity
FLcDNA: full-length cDNA
JA: jasmonic acid
LOX: lipoxygenase
MeJA: mehyl jasmonate
NLR: cytoplasmic nucleotide-binding domain and leucine-rich repeat
OPR: 12-oxo-phytodienoic acid reductase
PAL: phenylalanine ammonia lyase
PAMPs/MAMPs: pathogen- or microbe- associated molecular patterns
PBZ1: probenazole-induced protein 1
PDF: plant defensin
PR: pathogenesis-related
PTI/MTI: PAMPs/MAMPs-triggered immunity
qRT-PCR: quantitative reverse-transcription polymerase chain reaction
SA: salicylic acid
SAG: SA-O-β-D-glucoside
SAGT: SA glucosyltransferase
TAR: tryptophan aminotransferase of arabidopsis 1 (TAA1)-related
UPGMA: unweighted pair group method with arithmetic mean
VSP: vegetative storage protein
