# Supplementary material for: Expression profiling of marker genes responsive to the defence-associated phytohormones salicylic acid, jasmonic acid and ethylene in Brachypodium distachyon
Source: BMC Plant Biol. 2016 Mar 2;16:59. doi: 10.1186/s12870-016-0749-9 (PMC4776424; doi:10.1186/s12870-016-0749-9)
Supplement: Additional file 1: Figure S1. — Protein sequence alignments of OsWRKY45, BdWRKY45-1 and BdWRKY45-2 (PPTX 145 kb) [file 12870_2016_749_MOESM1_ESM.pptx]

## Slide 1
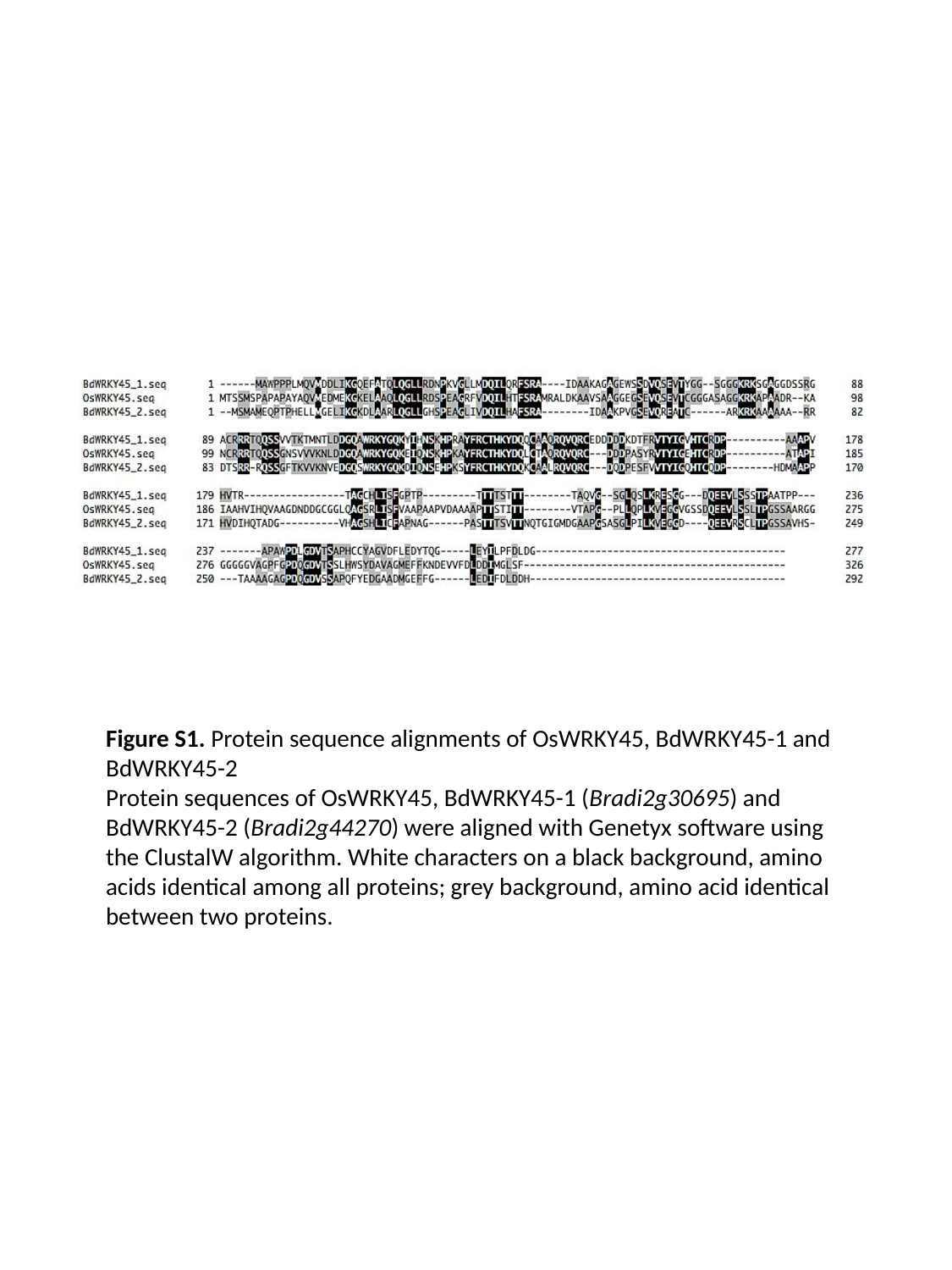

Figure S1. Protein sequence alignments of OsWRKY45, BdWRKY45-1 and BdWRKY45-2
Protein sequences of OsWRKY45, BdWRKY45-1 (Bradi2g30695) and BdWRKY45-2 (Bradi2g44270) were aligned with Genetyx software using the ClustalW algorithm. White characters on a black background, amino acids identical among all proteins; grey background, amino acid identical between two proteins.
