# Supplementary material for: Expression profiling of marker genes responsive to the defence-associated phytohormones salicylic acid, jasmonic acid and ethylene in Brachypodium distachyon
Source: BMC Plant Biol. 2016 Mar 2;16:59. doi: 10.1186/s12870-016-0749-9 (PMC4776424; doi:10.1186/s12870-016-0749-9)
Supplement: Additional file 2: Table S1. — Primers used in this study (DOCX 32 kb) [file 12870_2016_749_MOESM2_ESM.docx]

**Table S1.** Primers used in this study

| 1) Marker gene candidates | | | |  | 2) *BdPR1* genes | | | |
| --- | --- | --- | --- | --- | --- | --- | --- | --- |
|  | Gene | Name | Primer sequence |  | Gene | Name | Primer sequence |  |
|  | *Bradi2g05870* | NPR1-F | AGCTTCAACTCGACCAGCAT |  | *Bradi1g09637* | #1-F | TACTACACGCACGCCAACAA |  |
|  |  | NPR1-R | CGATCACCACATCATTGAGC |  |  | #1-R | GTCGTAGAGGCAGAGCGTG |  |
|  | *Bradi2g30695* | W45L1-F | GGACACCTTCAGGGTGACAT |  | *Bradi1g12360* | #2-F | CTACGACTACGACAGCAACA |  |
|  |  | W45L1-R | TTGTCGTCGTGGTAGGAGTG |  |  | #2-R | GGGTAGTAGTTGCAGGTGAT |  |
|  | *Bradi2g44270* | W45L2-F | GATCGGAGGTGCAGAGAGAG |  | *Bradi1g57540* | #3-F | AAGCTAGTTCGGTCCTTGGC |  |
|  |  | W45L2-R | GTGTGCACCGGAAGTAGGAT |  |  | #3-R | TCTCCCCGTATAAGGTCCCG |  |
|  | *Bradi4g35356* | SGT1-F | ACGCCAAGTACATCCAGGAC |  | *Bradi1g57580* | #4-F | GGAGAAGCGGTTCTACCACC |  |
|  |  | SGT1-R | CAGTCGATTGCTGGGATTTT |  |  | #4-R | GTCCATCGCCAGAGTCACAA |  |
|  | *Bradi2g22410* | AGA-F | TCCTGCTCTTTTGTCCGAGT |  | *Bradi1g57590* | #5-F | CAGGTGGTGTGGAGGAAGTC |  |
|  |  | AGA-R | TGTGATAAGCAGCGATGAGG |  |  | #5-R | CTCTCCCCGTTGAAGTTCCC |  |
|  | *Bradi1g53527* | 76L1-F | AGGTACGTGCAGGATGTGTG |  | *Bradi2g14240* | #6-F | TCTGGAACGACACACAGCTC |  |
|  |  | 76L1-R | TTGGTCCTCAATTCCTTTGC |  |  | #6-R | TTCTCCTTTCCAGTTCCCGC |  |
|  | *Bradi1g53540* | 76L2-F | CTCCACACCGGCTTCAAC |  | *Bradi3g53630* | #7-F | CAGTTCCTGTACCCGCAGAA |  |
|  |  | 76L2-R | GCGAGGATGATGGAGATGAT |  |  | #7-R | CTGCCCCAGAAGAGGTTCTC |  |
|  | *Bradi1g53550* | 76L3-F | AGTCTTCCCTCTCCCGTACC |  | *Bradi3g53637* | #8-F | GGAGAAGGCCATGTACGACG |  |
|  |  | 76L3-R | AAGATGTGCTCCACGGTCAC |  |  | #8-R | TAGTTGCCGGGCGGGTTATA |  |
|  | *Bradi4g41410* | 76L4-F | ATTCTCCTCTCCGCAACAGA |  | *Bradi3g60230* | #9-F | CCCTCCTTCTCCTCGCCTAT |  |
|  |  | 76L4-R | CGGTTAAGCTCCTGCTCTTG |  |  | #9-R | GGCGACAGAGACGTTGTACA |  |
|  | *Bradi1g11940* | 74L1-F | CGTCGAGGAGTACCTGGAGA |  | *Bradi3g60260* | #10-F | CTGTACTACCACAACGCGGT |  |
|  |  | 74L1-R | CCCTTCGTTCACGTAGTGGT |  |  | #10-R | GCCCCAGAAGATGTTCTCCC |  |
|  | *Bradi4g35350* | 74L2-F | AGAGGGATCGAGGAGGTGAT |  | *Bradi4g38910* | #11-F | GCTGTTCTACTTCGGCGAGA |  |
|  |  | 74L2-R | GAGTACTTGGCGACAAACTCG |  |  | #11-R | AGTTGCTGCCGTAGTCGTAC |  |
|  | *Bradi5g03380* | 74L3-F | CAGCCAACCACAGCAAAGTA |  |  |  |  |  |
|  |  | 74L3-R | GTCAGAGCTTCCTCCCTCCT |  |  |  |  |  |
|  | *Bradi1g69330* | AOS-F | ACCGCCTGGACTTCTACTAC |  |  |  |  |  |
|  |  | AOS-R | GAGGTTCTTCTTCTCCACCT |  |  |  |  |  |
|  | *Bradi1g11670* | LOX-F | TCAACTTGCCCTTTCCACATG |  |  |  |  |  |
|  |  | LOX-R | GCAAACCGGATTAACTCCTGC |  |  |  |  |  |
|  | *Bradi2g52370* | ERF-F | TTTTTCTACGAGCAGGCTAC |  |  |  |  |  |
|  |  | ERF-R | GTTCAGATCCAGATCAAACG |  |  |  |  |  |
|  | *Bradi1g63780* | EIN3-F | ATGCTGAATGACAAGTTCCT |  |  |  |  |  |
|  |  | EIN3-R | AGGTGTAGACACGGTTGTTC |  |  |  |  |  |
|  | *Bradi1g49966* | ACS-F | TTGCATTGAGCCTGGATGGT |  |  |  |  |  |
|  |  | ACS-R | TGTGGCTATGTGTGACCCTCCT |  |  |  |  |  |
|  | *Bradi2g34400* | TAR1L-F | GAATCGGGATGGTGGCCTCG |  |  |  |  |  |
|  |  | TAR1L-R | ATTGTCGGATCGCCGTGATC |  |  |  |  |  |
|  | *Bradi2g04290* | TAR2L-F | GGCTCCATACTACTCTTCGTATC |  |  |  |  |  |
|  |  | TAR2L-R | CAGTAGTAGGCCAGGTCGTG |  |  |  |  |  |
|  | *Bradi3g37300* | 4CL-F | GTTCGAGACGGTGAGGATGTTC |  |  |  |  |  |
|  |  | 4CL-R | CATACTTTCCAACAGCGTCGC |  |  |  |  |  |
|  | *Bradi3g48840* | PAL-F | TCTTTGAGGCAAACATTCTT |  |  |  |  |  |
|  |  | PAL-R | ATAGCAGCAGCCTCTATTTG |  |  |  |  |  |
|  | *Bradi1g33540* | PR5-F | ACGGCGTACTCGAAGCTGTT |  |  |  |  |  |
|  |  | PR5-R | CTACTGAAACACGGTGCCTC |  |  |  |  |  |
|  | *Bradi4g05040* | PR10-F | CTCACGGTGGAGTATGAGAG |  |  |  |  |  |
|  |  | PR10-R | CTCGACCTTCTTGAGCAAC |  |  |  |  |  |
